# Human CB1 Receptor Isoforms, present in Hepatocytes and β-cells, are Involved in Regulating Metabolism

**DOI:** 10.1038/srep33302

**Published:** 2016-09-19

**Authors:** Isabel González-Mariscal, Susan M. Krzysik-Walker, Máire E. Doyle, Qing-Rong Liu, Raffaello Cimbro, Sara Santa-Cruz Calvo, Soumita Ghosh, Łukasz Cieśla, Ruin Moaddel, Olga D. Carlson, Rafal P. Witek, Jennifer F. O’Connell, Josephine M. Egan

**Affiliations:** 1 Laboratory of Clinical Investigation, National Institute on Aging, National Institutes of Health, Baltimore, MD 21224, USA; 2 Department of Medicine, Johns Hopkins Medical Institutes, Baltimore, MD 21224, USA; 3 Thermo Fisher Scientific, 7300 Governor’s Way, Frederick, MD 21704 USA

## Abstract

Therapeutics aimed at blocking the cannabinoid 1 (CB1) receptor for treatment of obesity resulted in significant improvements in liver function, glucose uptake and pancreatic β-cell function independent of weight loss or CB1 receptor blockade in the brain, suggesting that peripherally-acting only CB1 receptor blockers may be useful therapeutic agents. Neuropsychiatric side effects and lack of tissue specificity precluded clinical use of first-generation, centrally acting CB1 receptor blockers. In this study we specifically analyzed the potential relevance to diabetes of human CB1 receptor isoforms in extraneural tissues involved in glucose metabolism. We identified an isoform of the human CB1 receptor (CB1b) that is highly expressed in β-cells and hepatocytes but not in the brain. Importantly, CB1b shows stronger affinity for the inverse agonist JD-5037 than for rimonabant compared to CB1 full length. Most relevant to the field, CB1b is a potent regulator of adenylyl cyclase activity in peripheral metabolic tissues. CB1b blockade by JD-5037 results in stronger adenylyl cyclase activation compared to rimonabant and it is a better enhancer of insulin secretion in β-cells. We propose this isoform as a principal pharmacological target for the treatment of metabolic disorders involving glucose metabolism.

The endocannabinoid system (ECS) is a key regulator of numerous physiological processes including food intake, energy balance and glucose metabolism^1^, and becomes over-activated in obesity and type 2 diabetes mellitus (T2DM)^2^,^3^. T2DM is characterized by hyperglycemia due to insulin resistance and pancreatic beta (β)-cell dysfunction. The cannabinoid 1 (CB1) receptor emerged as an attractive therapeutic target for obesity-related diseases since pharmacologic inhibition of CB1 receptor by inverse agonists such as rimonabant produced potent anorectic effects in both rodents and humans^4^,^5^. However, therapeutic use in whole populations revealed that rimonabant caused adverse psychiatric and neurological side effects such as irritability, anxiety and suicidal ideation^6^ and was therefore suspended from human use. Nevertheless, CB1 receptors are found in both central and peripheral tissues and several studies demonstrate beneficial effects on glucose metabolism and insulin sensitivity devoid of central CB1 receptor blockade and food intake^7^,^8^,^9^. Consequently, there is an interest in developing compounds that target peripheral CB1 receptors.

In rodent and human pancreatic islets of Langerhans an autonomous ECS exists, which includes the cannabinoid receptors and endocannabinoids (ECs) that are synthesized in a Ca^2+^- and glucose-dependent manner^2^,^10^,^11^,^12^. While the orphan cannabinoid receptor GPR55 has been reported in β-cells^13^,^14^, there is no consensus on which cell types within the islet express the CB1 or CB2 receptors^10^,^11^,^13^,^14^,^15^,^16^,^17^,^18^,^19^,^20^,^21^. The exact role(s) of the ECS in islets is also a point of contention. While some studies have reported that CB1 receptor stimulates insulin secretion^2^,^18^,^22^,^23^, others have reported the contrary^15^,^20^,^24^,^25^. Our previous work has demonstrated that first-generation CB1 receptor blockers, such as AM251 and rimonabant, stimulated β-cell proliferation both *in vitro* and *in vivo*^11^,^26^. Recently, a novel CB1 receptor inverse agonist with low brain penetrance named JD-5037 has been characterized^27^,^28^,^29^. This compound was shown to protect β-cell function by reducing islet inflammation and to restore glycemic plasma levels to normal in ZDF rats^30^. Moreover, JD-5037 treatment led to reduced body weight and hepatic steatosis, and reversed hyperleptinemia and insulin resistance in diet-induced obese mice^28^,^31^.

Despite the renewed interest in peripheral CB1 receptors, knowledge of their fundamental regulation and molecular signaling in humans is limited. Along with full length CB1 receptor, two additional N-terminal shorter isoforms are present in humans and not in rodents. These isoforms (CB1a and CB1b) result from intra-exonal splicing of the *CNR1* coding exon^32^,^33^,^34^. However, the pharmacological importance of these three different CB1 receptor isoforms has been subjected to debate^32^,^34^,^35^ and therefore their further characterization and respective actions may be of considerable interest. In the current study, we characterize the expression of the human CB1 isoforms in the brain and several peripheral tissues and determine their functional differences upon activation. CB1b is preferentially expressed in β-cells and hepatocytes, where it is a more powerful modulator of adenylyl cyclase activity than the other isoforms. We also demonstrate the therapeutic potential of the peripherally-restricted CB1 receptor blocker JD-5037 in human islets. These findings give rationale for the development of second generation selective CB1b receptor blockers aimed at regulating β-cell function and improving insulin sensitivity.

## Results

### Human CB1 receptor gene encodes unique isoforms which differ in potency of action

**Figure 1:**
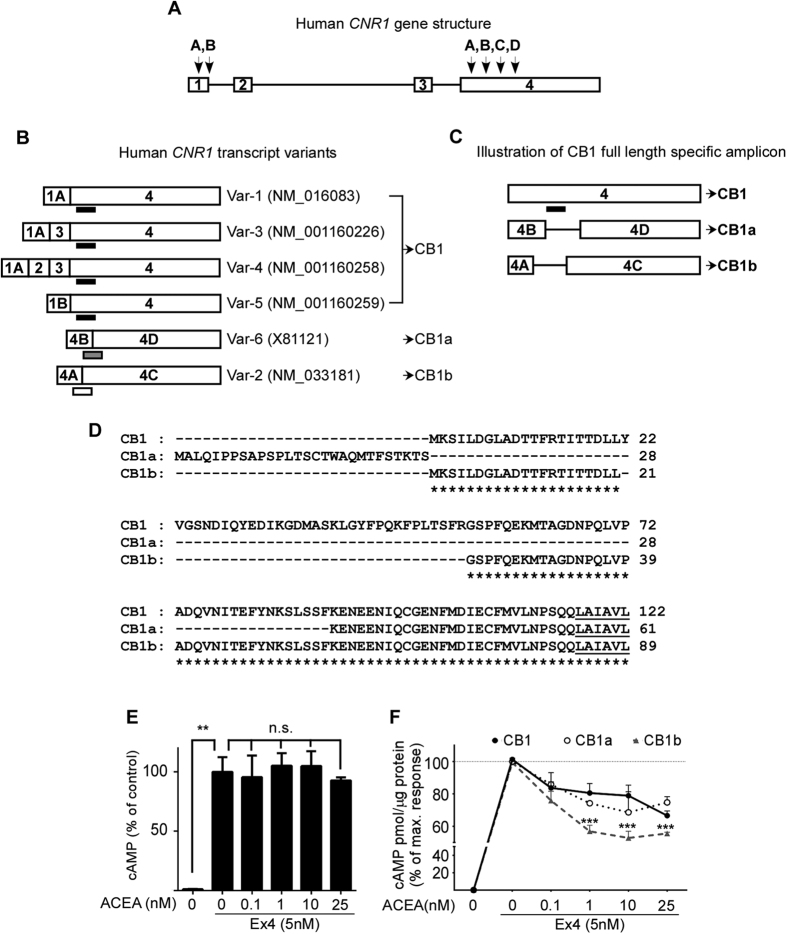
Human *CNR1* Gene Splice Variants. Human *CNR1* (6q15) gene structure (**A**) and its alternatively spliced transcript variants (**B**): the open bar represents the exons with exon numbers inside and horizontal lines represent the introns. Human *CNR1* has splicing sites in two of its exons (**A**). Within exon 1 there are two splicing sites (1A and 1B) and within coding exon 4 there are four splicing sites (4A-D) indicated by arrows above the exons. Alternatively-spliced transcript variants are shown (**B**) and GenBank accession numbers are included in parenthesis; the corresponding protein isoforms are indicated with an arrow. CB1 receptor full length protein and N-terminal isoforms are represented by CB1, CB1a and CB1b, respectively. CB1 full length amplicons align the common deleted region in between exon 4C-4D, recognizing all full length splice variants. Each TaqMan probe recognizes the adjacent and inter-exonal spliced exons and is represented by short horizontal bars in black, grey and white for CB1, CB1a and CB1b receptors, respectively. The specific full length CB1 receptor amplicon (black bar) aligns the intra-exonal sequence between splicing sites 4B and 4C, and does not recognize the spliced variants CB1a and CB1b (**C**). Alignment of the protein N-terminal amino acid sequences of CB1a and CB1b with full length CB1 receptor (**D**). Gaps are represented by dashed lines, identical amino acids by the asterisks. The beginning of the first transmembrane domain is underlined. Increasing concentrations of ACEA did not influence on Ex4-mediated cAMP accumulation in CHO cells when the cells were not transduced with any CB1 receptor (**E**). Cells were pre-treated with ACEA for 20 min before addition of Ex4 for a further 15 min. **p < 0.01, n.s. = not significant compared to vehicle. Treatment with the synthetic CB1 receptor agonist ACEA attenuated Ex4-mediated cAMP accumulation in CHO-GLP-1R cells when transduced with CB1 and CB1a, and to a greater extent with CB1b (**C**). All values were normalized to protein concentration. Data represent mean percentage over maximum response ± SEM from at least three independent experiments. ***p ≤ 0.005 compared to CB1 full length. For cell line validation, see Supplementary Fig. S1.

Human *CNR1* gene has four exons: within exon 1 there are two intra-exonal splicing sites (1A and 1B), and in coding exon 4 there are four intra-exonal splicing sites (4A-D), all of which are unique to humans (Fig. 1A). Alternative splicing of both exons 1 and 4 results in six transcript variants (Fig. 1B,C). Translation of intact exon 4 leads to the full length CB1 receptor protein, while intra-exonal alternative splicing events produce two N-terminal altered and deleted isoforms. CB1a lacks the first 89 amino acids of the full-length sequence in the N-terminus, and the sequence is substituted with 28 amino acids due to a frame-shift. CB1b has an internal deletion of 33 amino acids between Leu-21 and Gly-55 (Fig. 1D). Under high GLP-1R expression conditions, exendin 4 (Ex4) is a powerful activator of adenylyl cyclase and intracellular cAMP accumulation^36^. We therefore generated stable CB1-transfected CHO-GLP-1R cell lines expressing each isoform at a comparable level (Supplementary Fig. S1). Non-transfected CHO-GLP-1R cells lack CB1 receptors and the inhibition was not observed (Fig. 1E). Treatment of CHO-GLP-1R cells expressing full length CB1 receptor with the synthetic CB1 receptor agonist ACEA (≥1 nM) resulted in a 20% reduction of cAMP accumulation (Fig. 1F). This effect was further reduced by approximately 30% and 45% in the CB1a- and CB1b-expressing cells, respectively (Fig. 1F).

### N-terminal CB1 receptor isoforms are differentially expressed in human tissues

**Figure 2:**
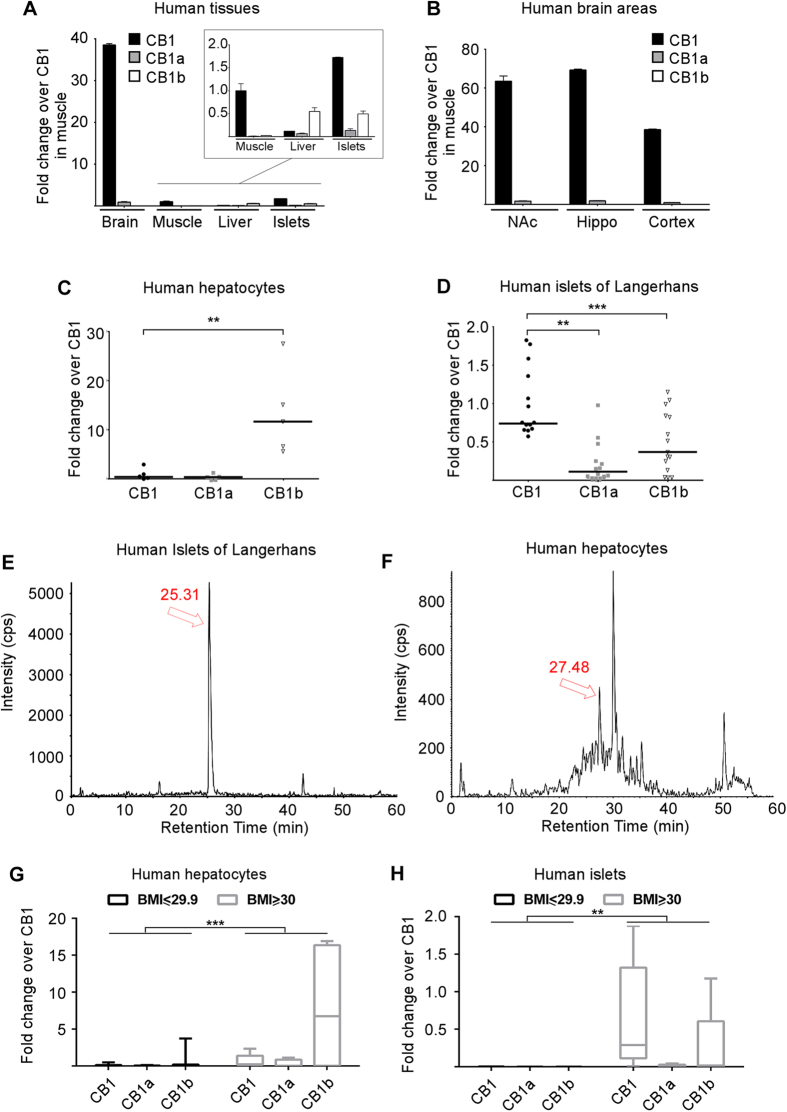
Relative abundance of CB1 receptor isoforms mRNA in human tissues. Real time PCR quantification of relative expression of CB1 receptor isoforms in brain and peripheral tissues (**A**). CB1 receptor isoforms expression in human brain regions (**B**): nucleus accumbens (NAc), hippocampus and cortex. Expression is represented as fold change over CB1 expression in muscle. Data show mean ± SD (n = 3); *p* ≤ 0.001. Real time PCR quantification of CB1 receptor isoforms in purified human hepatocytes (**C**) (n = 5 separate livers) and isolated islets of Langerhans (**D**) (n = 15 donors). Chromatographic separations of the digested protein samples for immunoprecipitated CB1 protein isoforms from human islets (43.1 BMI; confirmed T2DM donor) (**E**) and primary human hepatocytes (**F**). Isotopically labeled synthetic versions of the corresponding proteotypic peptides were used to determine retention times (Supplementary Fig. S3). Relative expression of CB1 isoforms in hepatocytes (**G**) or in isolated islets (**H**) from donors with a BMI lower than 30 (n = 17 for hepatocytes; n = 3 for islets) or ≥ 30 (n = 5 for hepatocytes; n = 5 for islets). Data represented as fold change over CB1, with horizontal bars representing the median; **p ≤ 0.01, ***p ≤ 0.001. β-actin was used as housekeeping gene.

Since CB1 receptor isoforms differ in their ligand binding and downstream signaling response^34^,^35^ (Fig. 1F), we sought to characterize the expression of these isoforms in human tissues. We first designed a combination of TaqMan primers and probes; the CB1a and CB1b amplicons align the intra-exonal junctions of exons 4B-4D and exons 4A-4C, respectively (Fig. 1B). Full length CB1 amplicons align the common deleted region between 4B-4C without interfering with CB1a or CB1b (Fig. 1C). As shown in Fig. 2A, the predominant isoform in the brain is full length CB1 with low relative expression of CB1a and virtual absence of CB1b. This pattern was consistent throughout the various brain regions tested (nucleus accumbens, hippocampus and cortex; Fig. 2B). Likewise, skeletal muscle displayed the same pattern of expression (Fig. 2A). Conversely, CB1b was clearly the predominant isoform in the liver, with a 10-fold greater expression compared to full length CB1 (Fig. 2A). To confirm that expression of the CB1 isoforms in liver was due to expression in hepatocytes specifically we analyzed primary human hepatocytes and found a predominance of CB1b (Fig. 2C). Within islets, full length CB1 was present along with high expression of the CB1b isoform, where levels were similar to those found in liver (Fig. 2A). To further confirm the expression in human islets, freshly isolated islets from 15 cadaveric donors were analyzed. The pattern of expression was the same as in whole islets: CB1b and CB1a expression levels were approximately 60% and 25% that of the full length, respectively (Fig. 2D). We confirmed the presence of CB1 protein in human islets from a T2DM donor using a targeted proteomic approach (Fig. 2E). Although we could not confirm CB1b protein in human islets, due to a paucity of tissue and its lower expression compared to CB1 within islets, CB1b protein was detectable in human hepatocytes (Fig. 2F). The overall expression of CB1 receptors was significantly higher in obese donors (BMI ≥ 30) compared to leaner donors in both hepatocytes (Fig. 2G) and islets (Fig. 2H).

### CB1 receptor isoforms are expressed in β-cells but not in α- or δ-cells in human islets

**Figure 3:**
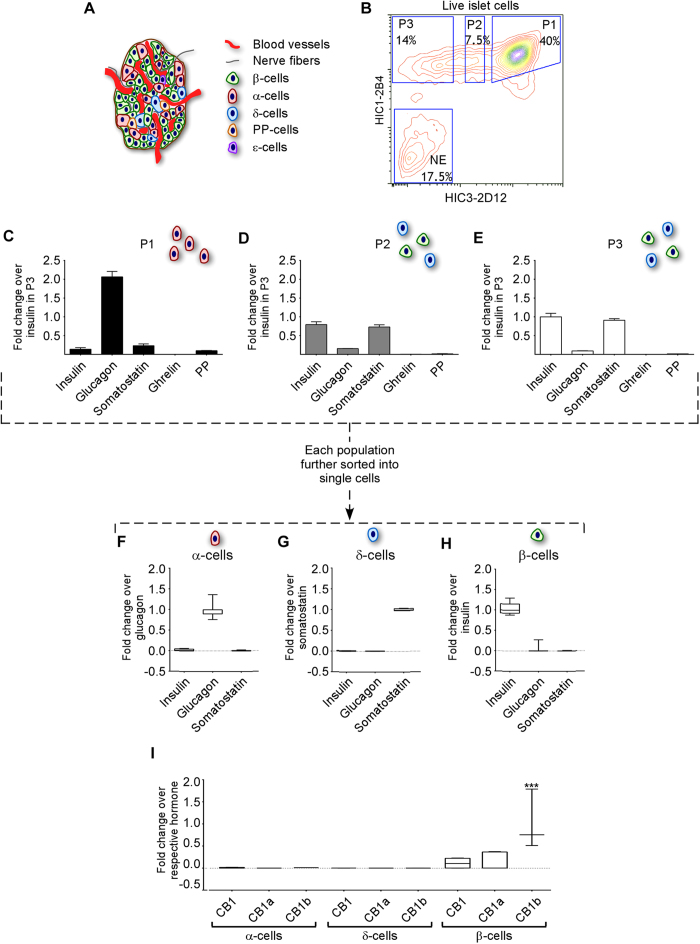
mRNA expression of CB1 receptor isoforms in single pancreatic β-cells. Islets of Langerhans are a complex cluster of different cell types: endocrine cells (α-, β-, δ-, ε- and PP-cells), nerve fibers and blood cells (**A**). Disaggregated human islets were stained with two antibodies for cell surface markers and sorted into three endocrine fractions (P1, P2 and P3) and a non-endocrine fraction (NE) (**B**). RNA from P1, P2 and P3 was extracted and retro-transcribed into cDNA. RT-PCR of the five endocrine hormones was performed to confirm the enrichment in each endocrine cell type (**C**–**E**). Expression was normalized to insulin in P3. Data represent mean ± SD, *p* < 0.001. The endocrine fractions (P1-P3) were then sorted into individual single cells. From those, RNA was extracted and reverse transcribed into cDNA, pre-amplified for the hormone transcripts of interest and diluted prior to real time PCR amplification. Expression was normalized to glucagon (**F**), somatostatin (**G**) and insulin (**H**). *p* ≤ 0.001. CB1 isoforms were quantified and represented as fold difference relative to corresponding hormone levels (**I**). ***p ≤ 0.005. Data are represented in box and whisker plot (n = 10). Relative amount of copies of each CB1 receptor isoform was quantified using specific Taqman primers and probes.

Islets are heterogeneous clusters consisting of five different endocrine cell types and other non-endocrine cells resulting from the natural innervation of the islet and its blood supply (Fig. 3A). To determine if the CB1 receptor isoforms were specific for β-cells, we separated β-cells from the other four endocrine cell types present within islets (namely glucagon-producing α-cells, somatostatin-producing δ-cells, pancreatic polypeptide-producing PP-cells, and ghrelin-producing ε-cells) using specific staining of disaggregated islets following cells sorting by FACS. Neither Newport Green™ DCF diacetate (NG) staining, which is based on the analysis of intracellular zinc content known to be enriched in insulin granules, nor targeting GLP-1R with FAM-Ex4, described as being specific for β-cells^37^,^38^ resulted in a pure β-cell population (Supplementary Fig. S4A–F). As an alternative approach, we utilized antibodies developed specifically for surface markers on human β-cells^39^. Human islets from a single cadaveric donor were dissociated and stained with HIC3-2D12 IgM and HIC1-2B4 IgG. The sorting resulted in four populations, P1, P2, P3, and non-endocrine cell population (NE) (Fig. 3B). While P1 was positive for glucagon expression and was highly enriched for α-cells (Fig. 3C), we were unable to obtain an equally enriched β-cell population by this method (Fig. 3D,E). We then further sorted these populations to obtain single cells. RNA from a single cell was equally divided into three aliquots and pre-amplified independently for each of CB1 receptor isoform-specific primers and probe set so as to avoid interference between the isoforms. The three major cell types were identified as α-, δ- and β-cells through expression of their specific hormones (Fig. 3C–E). We could not sort single PP- or ε-cells by FACS, presumably because of their low abundance in human islets. Only insulin-positive β-cells expressed the CB1 receptor, with predominant expression of CB1b, while CB1 receptor was not expressed in any other islet endocrine cell type examined (Fig. 3F).

### Activity of CB1 receptor inverse agonists is dependent upon human isoform expression

We next sought to determine if the two major isoforms present in pancreatic β-cells and hepatocytes, namely full length CB1 and CB1b, differed in their response to a global (rimonabant) versus peripherally-restricted (JD-5037) CB1 receptor inverse agonist. As a result, the binding affinity of rimonabant and JD-5037 was determined for both CB1 and CB1b receptors (Table 1). Rimonabant had a slightly stronger affinity (~3 fold) for the CB1 receptor, while JD-5037 affinity was significantly stronger (~30 fold) for the CB1b receptor (Table 1). Similar trends were observed with the functional assay, where JD-5037 inhibited CB1b receptor to a greater extent than CB1 (Supplementary Fig. S5). These data point at JD-5037 as a stronger inhibitor of the peripheral isoform CB1b than rimonabant.

**Figure 4:**
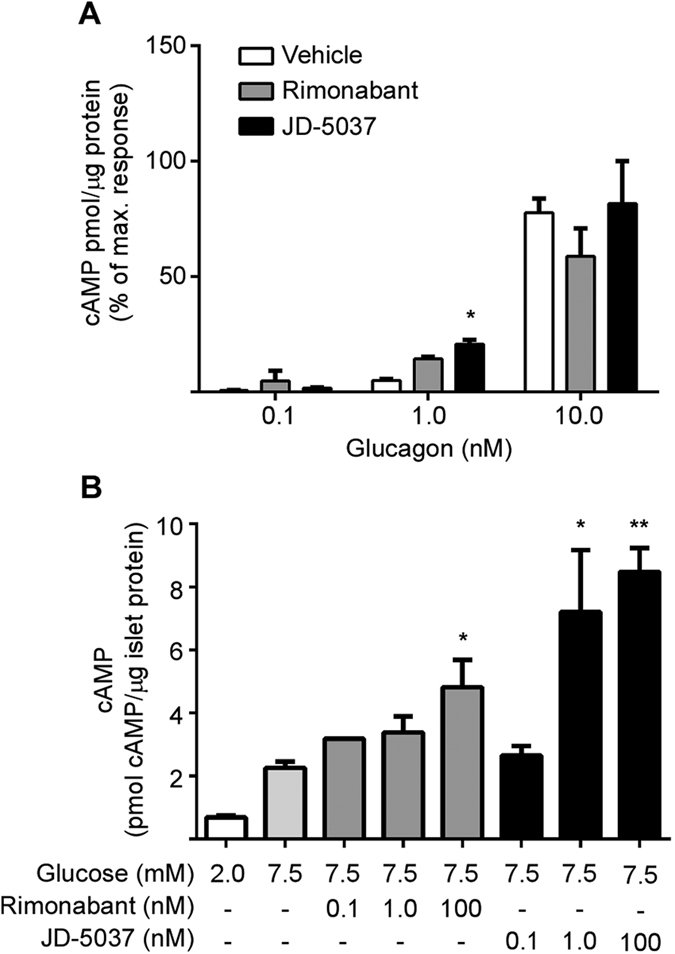
CB1 receptor human isoforms differ in activity for cannabinoid receptor inverse agonists in metabolic systems. Quantification of glucagon-stimulated cAMP accumulation in primary human hepatocytes (**A**). Hepatocytes from a human donor were pretreated with rimonabant (100 nM) or JD-5037 (100 nM) prior to glucagon stimulation. Data are the mean ± SEM from two independent experiments; *p ≤ 0.05 compared to vehicle. Quantification of cAMP accumulation in isolated human islets of Langerhans (**B**). Islets from one donor (20.4 BMI) were pre-cultured in 2 mM glucose for 2 hours followed by stimulation with 7.5 mM glucose in the presence or absence of increasing concentrations of rimonabant or JD-5037. Data are the mean ± SEM of 3 separated incubations; *p ≤ 0.05 compared to 7.5 mM glucose alone.

We have recently published that CB1 receptors influence adenylyl cyclase activity in β-cells in response to GLP-1^40^. In order to confirm that CB1b also regulates adenylyl cyclase activity that would lead to intracellular cAMP accumulation we utilized primary human hepatocytes, which endogenously express CB1b (Fig. 2C). Hepatocytes also express abundant glucagon receptors, which are G_s_ G-protein coupled receptors. Isolated primary human hepatocytes were treated with rimonabant or JD-5037 following glucagon stimulation. Blockade of the CB1b with JD-5037 significantly increased glucagon-mediated cAMP accumulation (Fig. 4A). In the presence of physiological concentrations of glucagon (1 nM), JD-5037 increased cAMP accumulation to a greater extent than rimonabant, while at low glucagon concentrations (0.1 nM) there was no significant effect. At supra-physiological concentrations of glucagon (10 nM), the cAMP amount reached a plateau and the effect of the inverse agonists was no longer evident. Similar data were obtained using primary hepatocytes from separate donors. In human islet of Langerhans, 1nM of JD-5037 significantly stimulated adenylyl cyclase activity (Fig. 4B). A concentration of up to 100 nM rimonabant was needed to obtain a significant increase on adenylyl cyclase activity (Fig. 4B).

### Peripherally-specific CB1 receptor blocker improves insulin secretion in human islets

**Figure 5:**
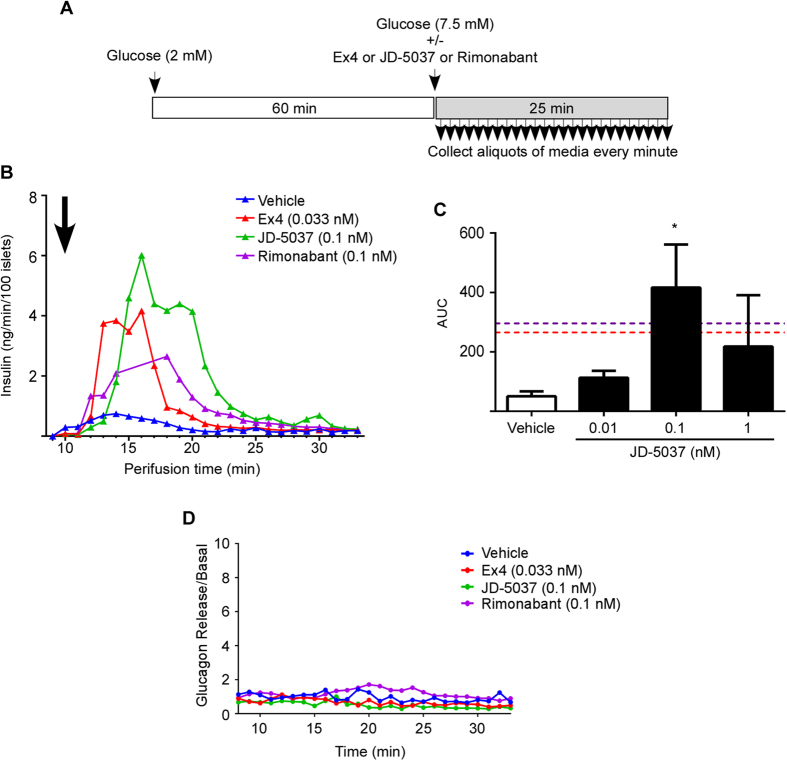
CB1 receptor blockade stimulates insulin secretion in isolated islets of Langerhans. Experimental timeline for perfusion of human islets (**A**). Insulin secretion from human islets perfused with glucose alone (7.5 mM) (blue line) and in combination with 0.33 nM Ex4 (red line) (half maximum insulin secretion), 0.1 nM JD-5037 (green line) or 0.1 nM rimonabant (purple line) (**B**). Treatment time point is indicated with a black arrow. Islets were obtained from a single obese donor (34.7 BMI). Area under the curve (AUC) of insulin secretion in perfused islets from 3 independent donors (BMIs of 53.2, 32.8 and 34.7) with glucose alone or in combination with increasing concentrations of JD-5037 (**C**). Ex4 and rimonabant AUC are indicated with red and purple dotted lines respectively. Glucagon secretion from human islets perfused with glucose alone, glucose and Ex4 or glucose and JD-5037 (**D**). Data are the mean ± SEM of 3; *p ≤ 0.05 compared to vehicle.

In order to assess if CB1b receptor blockade would directly influence insulin secretion, we used a perifusion system. Human islets from donors with BMI higher than 30 (when possible) were used since they have higher expression levels of CB1b receptor (Fig. 2F). Islets were first subjected to non-stimulatory levels of glucose (2 mM, equivalent to 36 mg/dl) in perfusion medium for 60 min (Fig. 5). We then increased the glucose level to 7.5 mM (135 mg/dl to mimic circulating blood level of glucose in non-diabetic subjects after an oral glucose tolerance test) in order to stimulate insulin secretion and EC synthesis (Fig. 5A). Islets that were perfused with 0.1 nM of JD-5037 in addition to stimulatory levels of glucose had a far greater increase in insulin secretion than islets subjected to glucose alone, Ex4 or to the same concentration of rimonabant (Fig. 5B). Stimulation of insulin secretion by JD-5037 was dose-dependent (Fig. 5C) and it had no effect on glucagon secretion (Fig. 5D).

## Discussion

The present study provides evidence that the human CB1 receptor isoform, CB1b, is expressed at significant levels in pancreatic β-cells. Furthermore, it is unequivocally the main isoform in hepatocytes: this has not been previously reported. We also report that JD-5037, a CB1 receptor inverse agonist with low brain penetrance^27^,^28^ shown to improve numerous metabolic parameters in rodent models of obesity^28^,^30^,^31^, has preferential inverse agonistic activity for CB1b when compared to full length CB1. Moreover, CB1b influences adenylyl cyclase activity in metabolically-active tissues and its blockade enhances insulin secretion in a glucose-dependent manner.

While it is accepted that CB1 receptors are present in human islets, controversy remains as to which cells within the islet express them^10^,^11^,^15^,^16^,^17^,^18^,^20^,^21^. The use of specific primers instead of CB1 receptor antibodies, which are fraught with non-specific binding^41^, combined with rigorous sorting of single islet cells allowed us to obtain a definitive answer. Here we confirm at the single cell level that in human islets the expression of this receptor is specific to β-cells, corroborating previous studies that the CB1 receptor is present in β-cells^11^,^18^,^21^,^22^, and is not found in the other major endocrine cell types. Human islets express both full length CB1 receptor and the CB1b receptor isoform at comparable levels, with little expression, if any, of CB1a. Upon islet disaggregation, isolated human β-cells actually mainly express the CB1b isoform. The difference in the pattern of expression between whole islets and isolated β-cells may be explained by the presence of nerves fibers and immune cells normally found in islet structures (Fig. 3A). Indeed, macrophages in islets have been shown to express their own ECS^30^. Furthermore, there is a positive relationship between CB1 isoforms expression and BMI in both isolated human hepatocytes and islets, in accordance to the previous literature that shows an overactivation of the ECS in obesity^2^,^3^, making this receptor an accessible target in obesity. The long N-terminal tail of the CB1 receptor can affect receptor trafficking to the membrane and therefore its availability and consequent activity^42^. In fact both shorter isoforms, CB1a and CB1b, have been shown to more potently regulate synaptic transmission in isolated hippocampal neurons^35^. In accordance, we demonstrate that the peripheral CB1b receptor isoform exhibits stronger inhibition of adenylyl cyclase than CB1 and CB1a. Therefore, its presence in human β-cells likely modulates the intensity of cAMP/PKA down-stream signaling. We found that JD-5037 has greater affinity for CB1b than CB1, which is not the case for other inverse agonists previously tested^32^,^34^, while rimonabant has greater affinity for full length CB1.

After proving the presence of CB1b in human hepatocytes by a proteomic approach and in order to study the potency of CB1b in modulating G_s_ G-protein activity, we treated primary human hepatocytes with CB1 receptor inverse agonists prior to stimulation of AC. Blockade by JD-5037 significantly enhanced glucagon-stimulated cAMP accumulation, confirming that CB1b executes a strong influence on adenylyl cyclase activity. In human islets of Langerhans stimulated with glucose, JD-5037 increased intracellular cAMP beyond that achieved by glucose and comparable to Ex4. Conversely rimonabant, less potent for CB1b inhibition, was needed at higher concentration to result in the same levels of cAMP as JD-5037. These results demonstrate that blockade of CB1b receptor strongly influences G_s_ activity in both hepatocytes and β-cells. While the activation of AC and resultant cAMP production in β-cells is well known to enhance insulin release, the influence of the CB1 receptor activation on insulin secretion is still hotly contested^2^,^15^,^18^,^20^,^22^,^23^,^24^,^25^. The existing discrepancies may be due in part to differences in the type of agonist/inverse agonists used, their concentrations, off-target effects and islet quality. Results are also affected by the design of the experiment (i.e. perifusion *vs*. static insulin release: static experiments result in accumulation of end products such as hormones and ECs) as well as differences caused by the use of cell lines that lack the islet’s multicellular and potentially neural and immune environment. In this study we have used human islets from healthy donors in a dynamic system stimulated with usual postprandial levels of glucose to stimulate both insulin secretion and EC synthesis, and perfused them with nanomolar concentrations of inverse agonists in order to avoid off target effects. We show that JD-5037 potently enhances insulin secretion from isolated human islets in a concentration-dependent manner. This degree of stimulation was donor-dependent, which may be a factor in reported discrepant findings. Furthermore, rimonabant stimulated insulin secretion to a lesser extent than JD-5037 when compared across various concentrations. The inverse agonist AM251 has been shown to stimulate insulin secretion in BRIN-BD11 cells, which is only partially reversed by the GPR55 antagonist cannabidiol^14^, possibly owing to a combination of GPR55 and CB1 targeting^29^. We conclude that in isolated islets, ECs play a role in dampening insulin secretion that is circumvented by a CB1b receptor inverse agonist. CB1b is therefore a possibility for therapeutic targeting of peripheral CB1 receptors. Future studies examining the mechanisms by which tissue-specific alternative splicing occurs as well as the relationship between isoform expression and pathological states such as T2DM and fatty liver states would enable greater understanding of peripheral CB1 receptor signaling.

In summary, our findings support that peripheral blockade of the CB1 receptor may result in beneficial effects on glucose homeostasis observed previously with globally-acting inverse agonists. We establish a novel target, CB1b, which is present in both liver and β-cells of humans, for possible therapeutic development.

## Methods

### Reagents

Arachidonyl-2-chloroethylamide (ACEA) was purchased from Cayman Chemical (Ann Arbor, MI). Ex4 was obtained from Bachem (Torrance, CA). JD-5037 and rimonabant were kindly donated by Drs. John F. McElroy and Robert Chorvat (Jenrin Discovery, Inc., West Chester, PA).

### Cell and islet culture

CHO cells and CHO-GLP-1R cells (CHO cells stably transfected with GLP-1R^36^) were maintained in DMEM/F-12 medium (Invitrogen, Grand Island, NY), 10% FBS, G418. CHO-GLP-1R cells were transduced with lentiviral particles for each of the CB1 isoforms^35^. Cells were plated on 24-well plates with regular media 24 h before transduction. Transductions were performed with 1 μl of Lentifect™ Lentivirus (Genecopoeia, Rockville, MD) and 8 μg/ml of Polybrene^®^ (Santa Cruz, Dallas, TX) for 24 h. Selection with 10 μg/ml of Puromycin (Invitrogen) was done 72 h after. Freshly isolated islets of Langerhans from human cadaveric donors were obtained from the NIH/NIDDK-supported Integrated Islet Distribution Program (http://www.iidp.coh.org). Islet were maintained in CMRL 1066 medium (Mediatech Cellgro, Herndon, VA), 5.6 mM glucose, 10% FBS, 100 μg/ml streptomycin and 100 U/ml penicillin in non-adherent flasks overnight prior to being processed. Primary human hepatocytes were obtained from GIBCO (Grand Island, NY) and plated at a density of 0.9 × 10^6 ^cells/ml onto 24-well collagen-I coated plates in plating medium (Williams E Medium supplemented with 5% FBS, 1 μM dexamethasone, 1% penicillin/streptomycin, 4 μg/ml bovine insulin, 2 mM GlutaMAXTM and 15 mM HEPES). Following a 5 h attachment period at 37 °C with 5% CO_2_, cells were washed briefly and incubated in serum-free medium (Williams E Medium supplemented with 0.1 μM dexamethasone, 0.5% penicillin/streptomycin, 6.25 μg/ml human insulin, 6.25 μg/ml human transferrin, 6.25 μg/ml selenous acid, 1.25 mg/ml BSA, 5.35 μg/ml linoleic acid, 2 mM GlutaMAXTM, and 15 mM HEPES) overnight.

### Assays for cAMP detection

CHO-GLP1R transduced cells were plated in complete media for 48 h. Cells were washed in PBS and pre-incubated for 2 h in serum-free media at 37 °C following pretreatment with DMSO or ACEA for 20 min before addition of 5 nM of Ex4 for a further 15 min. Primary human hepatocyte were plated and pretreated with DMSO, rimonabant or JD-5037 for 1.5 h at 37 °C before stimulation with various concentrations of glucagon (Eli Lilly and Company, Indianapolis, IN) for 5 min. Human islets of Langerhans were pre-cultured for 2 hours in 2 mM glucose insulin secretion assay buffer (130 mM NaCl, 5 mM KCl, 1 mM Na_2_HPO_4_-H_2_O, 1 mM MgSO_4_-7H_2_O, 2 mM CaCl_2_-2H_2_O, 20 mM HEPES, 25.5 mM NaHCO_3_, 0.1% BSA), following stimulation in 7.5 mM glucose insulin secretion assay buffer with DMSO (Vehicle), rimonabant or JD-5037 for 15 min in the presence of 25 μM 3-isobutyl-1 methylxanthine (Sigma-Aldrich, St Louis, USA) (non-specific phosphodiesterase inhibitor). Cells or islets were lysed in 0.1 M HCl for determination of cAMP by ELISA. cAMP was measured using the cAMP ELISA kit from Enzo Life Sciences (Farmingdale, NY) according to the manufacturer’s instructions. The data were normalized to protein concentration using a BCA protein assay (Pierce Biotechnology, Rockford, IL) or islet number, and estimated from a minimum of three independent experiments, each performed in triplicate.

### Isolation of β-cells population by FACS

Islets (10,000) were picked manually, washed in DPBS (PBS 1X, 0.1% HAS, 2 mM MgCl_2_) and dissociated with TrypLE (Invitrogen) at 37 °C for 15 min. The reaction was stopped with cold FBS and cells were filtered through a 40 μm filter and washed in DPBS. A β-cell enriched population was sorted using cell surface markers developed by Markus Grompe^39^. HIC3-2D12 IgM and HIC1-2B4 IgG were added at 1:20 for 30 min on ice, washed with DPBS, and incubated for 30 min on ice with secondary antibodies 1:200 (R-Phycoerythrin conjugated Anti-Mouse IgM, Alexa Fluor^®^ 488 conjugated Anti-Mouse IgG; Jackson ImmunoResearch). Prior to sorting, cells were washed, filtered and resuspended in fresh media. For dead cell discrimination, cells were stained with Violet LIVE/DEAD^®^ Fixable Dead Cell Stain Kit, following the manufacturer’s instructions (Molecular Probes, Grand Island, NY). Dissociated and stained islet cells were analyzed and sorted using a FACSAria I flow cytometer/cell sorter (BD Biosciences, San Jose, CA). All sorted cells were subjected to doublet discrimination gating strategy and dead cell discrimination.

### Isolation of single β-cells by FACS

Single cell sorting was performed using the automated cell deposition unit (ACDU). Analysis of flow cytometry data was performed using FCS Express software (De Novo software). Sorted cells were collected directly from FACS into 50 μl of extraction buffer from PicoPure RNA Isolation Kit (Invitrogen) and mRNA extraction was performed following manufacturer’s protocol. Whole mRNA extracts were converted to cDNA with SuperScript III (Invitrogen). Single cells cDNA samples were divided equally in three. Pre-amplification of cDNA was carried out with TaqMan PreAmp Master Mix (Applied Biosystems, Carlsbad, CA) in a 14 cycle PCR^43^,^44^. For semi-qPCR, 2 μl of a 1:3 dilution was used with TaqMan Fast Advanced Master Mix (Applied Biosystems). Samples with unequal distribution of cDNA (assessed by β-actin expression) were discarded.

### Real Time PCR analysis

Human brain and peripheral tissue cDNAs were synthesized using pooled total RNA purchased from Clontech (Palo Alto, CA). Total RNAs were purified from whole islets or sorted islet cells (500–2000 cells) using Trizol Reagent (Invitrogen) and RNeasy Plus Mini Kit (Qiagen, Valencia, CA). Reverse transcription was performed with SuperScript III First-Strand Synthesis System (Invitrogen). Gene expression was assayed by qPCR with TaqMan Fast Advanced Master Mix (Applied Biosystems). Primers and FAM-labeled TaqMan probes were ordered from Applied Biosystems (the sequences of custom primers and probes are provided in Supplementary Table S1). Human VIC-labeled β-actin (ABI Cat#43263115E) was used for endogenous control of the duplex assay. Custom primers and probes were designed with Primer Express v3.0 (Applied Biosystems) and validated by CHO transduction with the CB1 isoforms. Efficiency testing was performed and was equal for all CB1 isoform primers and probes in comparison with β-actin endogenous control (Supplementary Fig. S2).

### Detection of CB1 protein by Chromatographic Analysis

CB1 isoforms immunoprecipitation from CHO-CB1 cells, CHO-CB1b cells, human primary hepatocytes and human islets using Anti-CB1 Monoclonal Antibody (ImmunoGenes, Switzerland) and proteomic analysis of CB1 isoforms were carried out as described previously (Ghosh et al, unpublished material).

### CB1/CB1b receptor binding and functional assay

The receptor binding assay in the column was carried out as previously described^45^ utilizing CHO cells stably expressing CB1 receptor isoforms. Briefly, 0.5 nM [^3^H]-CP55,490 (Perkin Elmer, Waltham, MA) was used as the marker ligand. A series of concentrations of the displacer ligands were added with the marker ligand: CB1: JD-5037: 0.25nM-100 nM and rimonabant 0.25 nM-10 nM; CB1b: JD-5037: 0.2–10 nM and rimonabant: 0.5–100 nM. The CB1/CB1b receptor plate-based functional assay was performed following a previously described protocol^29^ utilizing CHO-GLP-1R cells stably expressing CB1 receptor isoforms.

Plate based functional assay was performed in CHO cells stably expressing CB1 or CB1b were cultured in a 96-well plate in DMEM/F12, 10% FBS. After 48 h, when cells reached 100% confluence, media was changed for fresh one containing serial dilutions of the inverse agonist. Each experiment was run using a 1:4 serial dilution starting from 4 μM. Six replicates were performed on every plate for each concentration. After 1 h at 37 °C, the radioactive CB1 receptor agonist [^3^H]CP55,490 (Perkin Elmer, Waltham, MA) was added at a final concentration of 2 nM and incubated for 45 min at 37 °C. Supernatants were discarded and cells washed twice with PBS. Cells were lysed with NaOH (1 M). Lysates were transferred to scintillation vials containing scintillation liquid (National Diagnostics, Charlotte, GA). Data were analyzed with nonlinear regression and the log(inhibitor) vs. response (three parameters) curve using GraphPad Prism software.

### Human islet perifusion

Human cadaveric islets perifusion experiments were performed using a mini-perifusion system previously described^46^. Islets were cultured overnight prior to use in the assay. Approximately one hundred islets were placed into insulin secretion assay buffer in polyacrylaminde P4 BioGel fine (Biorad, Hercules, CA). The islet-gel mixture was placed into the perifusion column (Biorep Technologies, Miami, FL) and connected to the perifusion system. Islets were perifused for 1 h in low glucose (2 mM) at a rate of 100 μl/min, followed by perifusion with either stimulatory glucose (7.5 mM) alone or in combination with Ex4, rimonabant or varying concentrations of JD-5037 for 25 min. During this stimulatory period, samples were collected every min. The dead volume of the perifusion system is 11 min. Insulin concentrations were determined by ELISA (Mercodia, Uppsala, Sweden) according to the manufacturer’s instructions.

## Additional Information

**Accession codes:** GenBank Accession: KC292264.

**How to cite this article**: González-Mariscal, I. *et al.* Human CB1 Receptor Isoforms, present in Hepatocytes and β-cells, are Involved in Regulating Metabolism. *Sci. Rep.* **6**, 33302; doi: 10.1038/srep33302 (2016).

## Supplementary Material

Supplementary Information

## Figures and Tables

**Table 1:** Central and peripheral CB1 receptor human isoforms differ in affinity for cannabinoid receptor inverse agonists.

Ligand	Receptor Isoform	
	CB1 (nM)	CB1b (nM)
Rimonabant	2.30 ± 0.69	7.65 ± 3.79
	(R^2^ = 0.973)	(R^2^ = 0.984)
JD-5037	8.16 ± 1.51	0.24 ± 0.12
	(R^2^ = 0.994)	(R^2^ = 0.824)

The Ki of rimonabant and JD-5037 for the CB1 and CB1b receptors as determined on the CHO-CB1 and CHO-CB1b open tubular columns, respectively, using frontal affinity chromatographic techniques.
