# Supplementary material for: Human CB1 Receptor Isoforms, present in Hepatocytes and β-cells, are Involved in Regulating Metabolism
Source: Sci Rep. 2016 Sep 19;6:33302. doi: 10.1038/srep33302 (PMC5027555; doi:10.1038/srep33302)
Supplement: Supplementary Information [file srep33302-s1.doc]

**Human CB1 Receptor Isoforms, present in Hepatocytes and β-cells, are Involved in Regulating Metabolism**

Isabel González-Mariscal^1^, Susan M. Krzysik-Walker^1^, Máire E. Doyle^2^, Qing-Rong Liu^3^, Raffaello Cimbro^2^, Sara Santa-Cruz Calvo^1^, Soumita Ghosh^1^, Łukasz Cieśla^1^, Ruin Moaddel^1^, Olga D. Carlson^1^, Rafal P. Witek^4^, Jennifer F. O'Connell^1^, Josephine M. Egan^1^

**SUPPEMENTARY METHODS AND DATA**

**Isolation of a pure β-cell population by FACS**

A β-cell enriched population was sorted from other islet cells using either a zinc staining dye, Newport Green™ DCF diacetate (NG) (Invitrogen), or fluorescein-exendin 4 (F-Ex4) (Anaspec, Fremont, CA). Briefly, NG was prepared at 1 µM and F-Ex4 at 10 nM, and the staining performed for 30 min at 37°C. Disaggregated islets were washed with PBS containing 0.1% Human Serum Albumin (Invitrogen) and resuspended in DPBS.

**Statistical analysis**

Data were represented as the mean or the median of the values ± SD or SEM, and analyzed using GraphPad Prism software (version 6.01; GraphPad Software, Inc., La Jolla, CA). Statistical analyses were performed using either an analysis of variance (ANOVA) with Bonferroni’s post hoc test analysis, student’s t-test for unpaired data (IC_50_ analysis), or Kruskal-Wallis (for non-parametric data; cell signaling analysis). Differences were considered statistically significant at *p* values < 0.05.

**
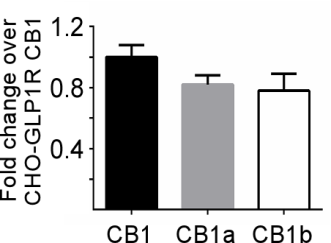
**

**Supplementary Figure S1.** CB1 splice variants mRNA levels in CHO-GLP-1R cells infected with lentiparticles of each CB1 isoform. mRNA levels were normalized to β-actin. Data show mean ± SD (n=3).


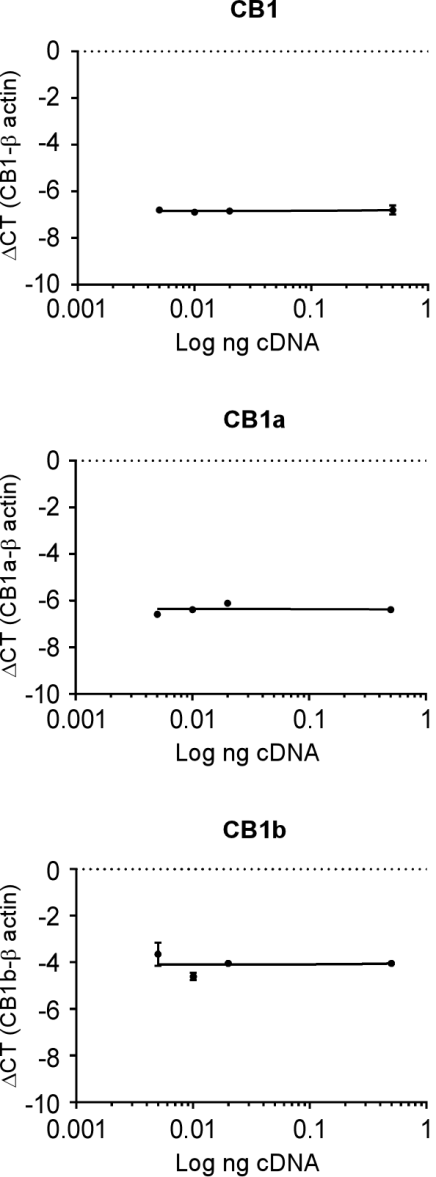


**Supplementary Figure S2.** Relative efficiency test for human CB1 isoform and endogenous control β actin: human nucleus accumbens cDNA was serially diluted to 0.5, 0.02, 0.01, 0.005 ng/ml and spiked with CB1, CB1a and CB1b plasmid, respectively, with the same serial dilutions. Duplicate TaqMan assay was performed using FAM-labeled CB1 isoforms and VIC-labeled actin βin TaqMan Fast Instrument with the default cycling program. The graphs were plotted Y as ΔCT of CB1 – β actin and X as log10 concentrations of cDNA-plasmids. The slopes of linear regression are <0.1, indicating the relative equal amplification efficiency of the target and the reference control.

**
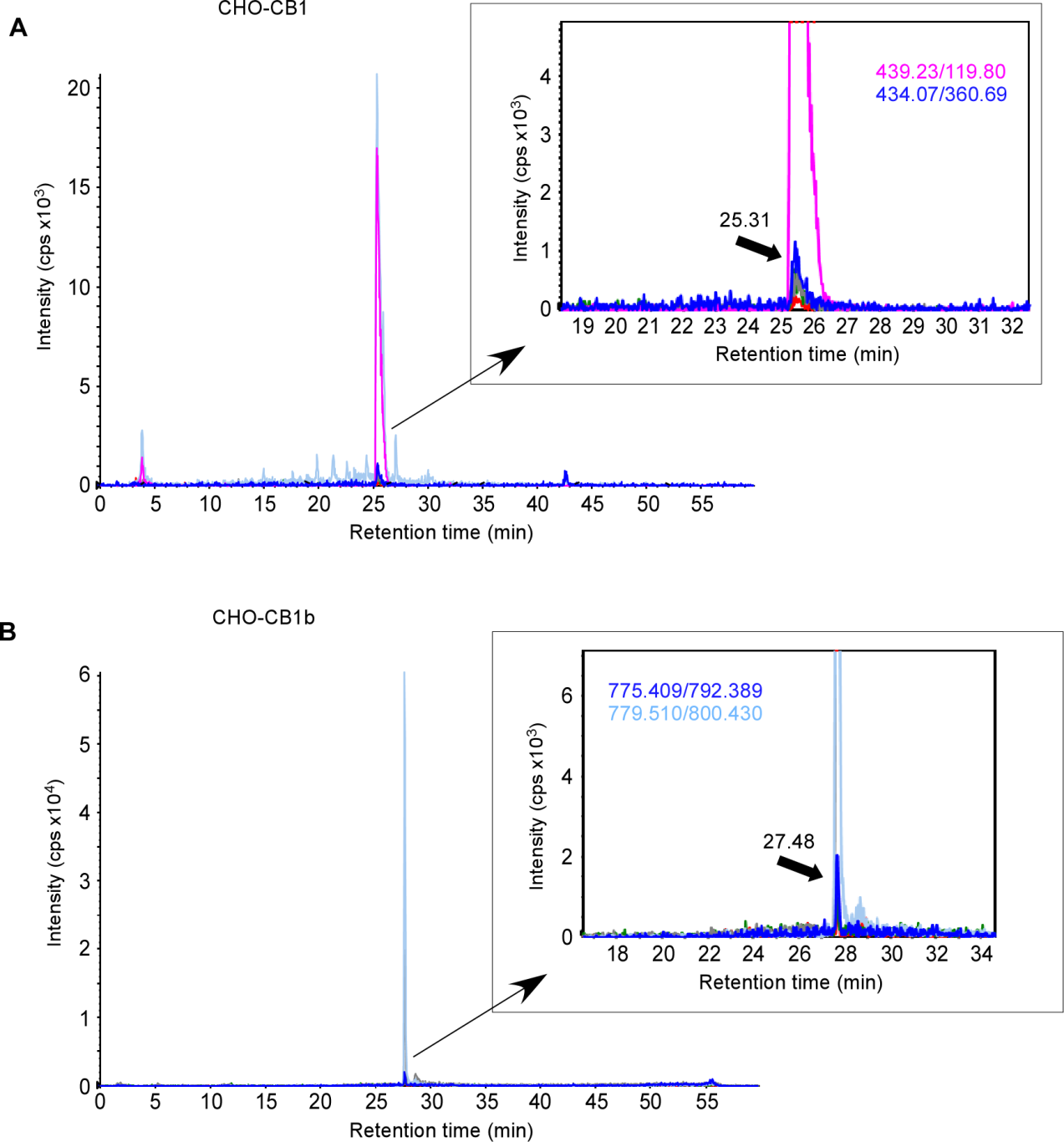
**

**Supplementary Figure S3.** Chromatographic traces of the digested protein samples from immunoprecipitated CB1 protein isoforms from **A**. CHO-CB1 cells containing the CB1 proteotypic peptide (FPLTSFR) (blue) and **B**. CHO-CB1b cells containing the CB1b proteotypic peptide (TITTDLLGSPFQEK) (blue) and their co-eluting heavy isotopically labeled synthetic versions (pink and light blue respectively).

**
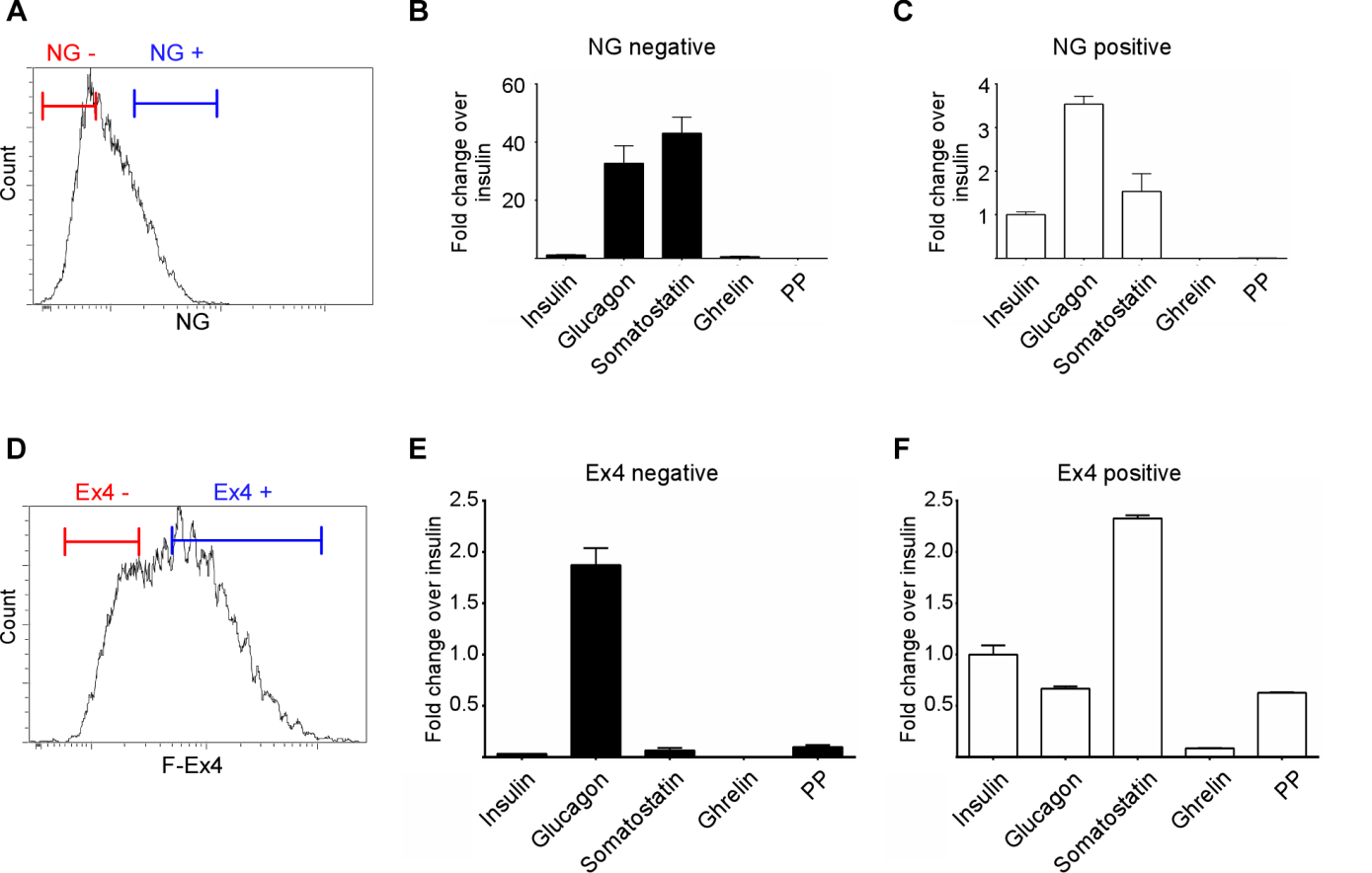
**

**Supplementary Figure S4. RT-PCR analysis after cell staining with antibody, NG or F-Ex4 and cell sorting by FACS of human disaggregated islets.** Islets were disaggregated and stained with Newport Green™ DCF diacetate (NG) (*A*) or with Fluorescein-Exendin 4 (F-Ex4) (*D*) and sorted into two populations, fluorophore negative (-) (*B*, *E*) and fluorophore positive (+) (*C*, *F*). RNA was extracted and reverse transcribed into cDNA, and RT-PCR of the five endocrine hormones performed. Expression is represented as fold changes from insulin. Data represent mean ± SD, *p*<0.001.

**
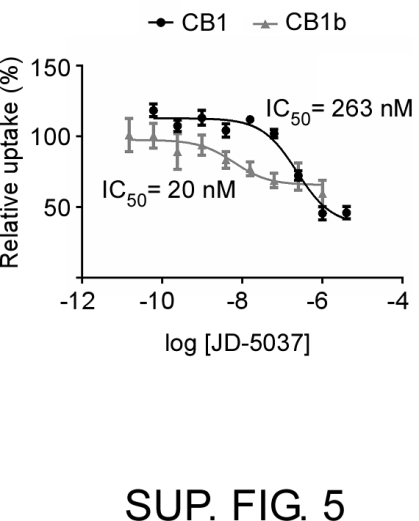
**

**Supplementary Figure S5.** **Central and peripheral CB1 receptor human isoforms differ in affinity for JD-5037.** Dose-response curve of JD-5037 on the inhibition of [^3^H]CP55,490 incorporation in CHO cells stably transduced with CB1 or CB1b isoforms to give equal expression. IC_50_ represents the mean of 3 independent experiments.

**Table S1. Taqman Primers and Probe sets for CB1 receptor isoforms.**

|  | | | |
| --- | --- | --- | --- |
| Gene | 5’-3’ Forward Primer | 5’-3’ Reverse Primer | Probe |
| CB1 | CCTGTACGTGGGCTCAAATGA | ACCCTAATTTGGATGCCATGTC | ATTCAGTACGAAGACATCA |
| CB1a | GGCTCAAATGACATTCAGTACGAA | GGACCATGAAACACTCTATGTCCAT | ACATCAAAGGAGAATGAGGA |
| CB1b | GATACCACCTTCCGCACCAT | CCGCAGTCATCTTCTCTTGGA | ACTGACCTCCTGGGAAG |
